# University students’ awareness of causes and risk factors of miscarriage: a cross-sectional study

**DOI:** 10.1186/s12905-018-0682-1

**Published:** 2018-11-19

**Authors:** Indra San Lazaro Campillo, Sarah Meaney, Jacqueline Sheehan, Rachel Rice, Keelin O’Donoghue

**Affiliations:** 1 Pregnancy Loss Research Group, The Irish Centre for Fetal and Neonatal Translational Research, University College Cork, Cork, Ireland; 2 National Perinatal Epidemiology Centre, Department of Obstetrics and Gynaecology, University College Cork, 5th floor, Cork University Maternity Hospital, Wilton, Cork, T12 YE02 Ireland

**Keywords:** Miscarriage, University students, Awareness, Prevalence, Risk factors

## Abstract

**Background:**

Spontaneous miscarriage is the most common complication of pregnancy, occurring in up to 20% of pregnancies. Despite the prevalence of miscarriage, little is known regarding peoples’ awareness and understanding of causes of pregnancy loss. The aim of this study was to explore university students’ understanding of rates, causes and risk factors of miscarriage.

**Methods:**

A cross-sectional study including university students. An online questionnaire was circulated to all students at the University College Cork using their university email accounts in April and May 2016. Main outcomes included identification of prevalence, weeks of gestation at which miscarriage occurs and causative risk factors for miscarriage.

**Results:**

A sample of 746 students were included in the analysis. Only 20% (*n* = 149) of students correctly identified the prevalence of miscarriage, and almost 30% (*n* = 207) incorrectly believed that miscarriage occurs in less than 10% of pregnancies. Female were more likely to correctly identify the rate of miscarriage than men (21.8% versus 14.5%). However, men tended to underestimate the rate and females overestimate it. Students who did not know someone who had a miscarriage underestimated the rate of miscarriage, and those who were aware of some celebrities who had a miscarriage overestimated the rate. Almost 43% (*n* = 316) of students correctly identified fetal chromosomal abnormalities as the main cause of miscarriage. Females, older students, those from Medical and Health disciplines and those who were aware of a celebrity who had a miscarriage were more likely to identify chromosomal abnormalities as a main cause. However, more than 90% of the students believed that having a fall, consuming drugs or the medical condition of the mother was a causative risk factor for miscarriage. Finally, stress was identified as a risk factor more frequently than advanced maternal age or smoking.

**Conclusion:**

Although almost half of the participants identified chromosomal abnormalities as the main cause of miscarriage, there is still a lack of understanding about the prevalence and most important risk factors among university students. University represents an ideal opportunity for health promotion strategies to increase awareness of potential adverse outcomes in pregnancy.

**Electronic supplementary material:**

The online version of this article (10.1186/s12905-018-0682-1) contains supplementary material, which is available to authorized users.

## Background

Miscarriage is one of the most common complications in pregnancy [1]. It is estimated that one out of four clinically recognised pregnancies will end in miscarriage during the first-trimester, and approximately 1% of pregnant women will experience a second-trimester miscarriage [2]. Despite the prevalence of miscarriage, 50% are attributed to chromosomal abnormalities [3], and a considerable percentage are classified as unexplained [4]. Therefore, identifying risk factors and effective interventions to prevent miscarriage has become a priority in the medical and scientific community [5]. Well-known risk factors include advanced maternal and paternal age, heavy smoking, alcohol consumption, infertility and previous miscarriage [6–10].

Preconception health care aims to identify and increase awareness to reduce risk factors before pregnancy that might affect the future maternal, child and family health [11–13]. An effort has been made to develop effective intervention plans and to include preconception risk factors in prenatal prevention programs internationally [14–18]. One of the main recommendations is to promote effective preconception health care interventions to develop curricula of preconception risk factors at undergraduate and postgraduate level [15]. Insight into students’ awareness of miscarriage might help to assess the effectiveness of preconception care education at a university level, but also to highlight the gaps of knowledge among this targeted population. Therefore, a cross-sectional study was conducted to explore university students’ understanding of prevalence, causes and risk factors of miscarriage.

## Methods

### Study design and data source

A cross sectional study was carried at University College Cork (UCC). Cork is one of the three cities in the Republic of Ireland with the highest full-time enrolments in the academic year 2016/2017 [19]. UCC currently has 20,000 full-time students of whom 14,000 are undergraduate [19]. It has over 3000 international students from 100 countries around the world. There are over 120 degree and professional programmes in Medicine, Dentistry, Pharmacy, Nursing and the Clinical Therapies, along with the Humanities, Business, Law, Architecture, Science, Food and Nutritional Sciences, available at UCC. Students were asked to select their area of study at UCC from a list of six options. For the purpose of this study, this list was grouped into four categories in accordance with the organisation of the Colleges within the University (i.e. The College of Medicine and Health, The College of Arts and Social Science, The College of Engineering & Food Science and The College of Business and Commerce & Law) [20]. For example, the College of Medicine and Health includes the Schools of Medicine, Dental School, Clinical Therapies, Nursing and & Midwifery, Pharmacy and Public Health.

An online questionnaire was circulated to all students at UCC using their university email accounts, in April and May 2016. The questionnaire was compiled using SurveyMonkey®, which is a user-friendly site to develop and administer online surveys. The questionnaire was anonymous and voluntary. An informed consent form explaining the objectives of the survey had to be completed before accessing the questionnaire. The main questionnaire consisted of twenty-six questions utilised to assess students’ understanding of the topic of miscarriage. Topics included general demographic and educational characteristics (i.e. sex, age, marital status, discipline and level of study), general knowledge and risk factors for miscarriage (i.e. agree, disagree and unsure of both well-known and spurious risk factors), identification of previous experience of miscarriage among themselves or their partners, and awareness of family member, friends or a celebrity who had a miscarriage. Students were asked to select the most common causes of miscarriage from a list of six options including lifestyle of mother (i.e. smoking and alcohol), medical condition or medical problem with the mother; genetic problem with the baby; psychological problems during pregnancy (i.e. stress, depression) and incident during pregnancy (i.e. fall, injury, accident). In addition, students were asked to provide rates of miscarriage in Ireland (i.e. “*In your opinion, what percentage of pregnancies in Ireland ends in a miscarriage? Please insert a number anywhere from 0 to 100 %”)* and weeks of gestation at which miscarriage occurs (“*when can a miscarriage occur? Between week “x” to week “x” of a pregnancy*”).

Definitions of miscarriage vary significantly between countries and jurisdictions [21]. For the purposes of this study, miscarriage is defined as the spontaneous demise of a pregnancy from the time of conception up to 24 completed weeks of gestation [22–24]. This study also reported the number of students who were only aware of first trimester miscarriage, which is defined as the loss of a pregnancy up to 12 weeks of gestation [22–24]. It is estimated that approximately one fifth of clinical pregnancies will end in a miscarriage in Ireland [24]. Therefore, a rate of 20% of miscarriage was selected as the cut-off rate in this study.

### Statistical analysis

Descriptive analysis was carried out using mean and standard deviation (SD) for continuous variables and percentages for categorical variables. Age was categorised using tertiles (i.e. 33.3% of the students were 21 years old or younger and 66.7% were 23 years old or younger). Three categories were created to calculate the number of students who underestimated (i.e. below the correct answer), correctly estimated or overestimated (i.e. above the correct answer) the rate of miscarriage. Information regarding the university students’ knowledge about contributory risk factors of miscarriage was assessed using a 5-point Likert scale ranging from strongly agree to strongly disagree. In the context of this study, answers were categorised as agree, unsure and disagree.

Chi-square tests were performed to assess the relationship between general demographic and educational characteristics, and knowing someone who had a miscarriage and identifying the correct rate of miscarriage. Chi-square tests were also calculated to investigate the relationship between independent variables and awareness of the most common causes of miscarriage. Binary logistic regression was calculated to estimate the probability of selecting risk factors for miscarriage (i.e. agree versus disagree) and general demographic and educational characteristics, knowing or not someone who had a miscarriage (i.e. themselves, partners, family, friends or celebrities) and whether the rate of miscarriage was correct, underestimated or overestimated. A high number of university students were unsure of their answers, and therefore, we also explored the relationship between agree versus unsure in the identification of risk factors for miscarriage; however, only those results which showed statistically significant differences and which added extra information to the comparison were reported.

A total number of 25 possible causes of miscarriage were alphabetically ordered in the questionnaire. For the purpose of this study we only analysed the Odds Ratios for those risk factors with a strong association with miscarriage (i.e. age, chromosomal abnormalities, smoking, alcohol and medical condition of mother) and for some spurious risk factors for miscarriage (i.e. flu vaccine, flying, hair dye, verbal arguments and vitamin C). Unadjusted and adjusted odds ratios (OR and aOR respectively) were calculated for all independent variables with their corresponding 95% confidence intervals (CI). All the analyses were performed using SPSS 21.0 (IBM).

## Results

Overall, 872 students responded to the online survey. Of those, 126 were excluded from the analysis because they did not complete more than half of the survey or they had highly extreme answers in demographic characteristic such as age. Therefore, a total sample of 746 university students were included in our analysis. The mean age was 24.3 years (SD = 6.58), and most of students were between 21 and 22 years old (*n* = 284; 38.1%) or were 23 years old or older (*n* = 289; *n* = 38.7%) ranging between 18 and 60 years old. More than half of the respondents were females (*n* = 577; 77.3%), and approximately 80% were single (*n* = 617). The discipline with the lower response rate was Business and Commerce and Law (*n* = 104; 13.9%) and with the highest response rate was Medicine and Health (*n* = 280; 31.9%).

Male students were more likely to report that they did not know anyone who had a miscarriage compared to female students (23.9% versus 9.6%; *p* < 0.001). Students aged 23 years old or older were more likely to report they knew someone who had a miscarriage; however, students of 20 years of age or younger were more likely to report they were aware of a celebrity who had had a miscarriage (*p* < 0.05). Single students were also more likely not to know anyone who had a miscarriage compared to those who had a partner, were married, were cohabiting or divorced (14.1% versus 5.8%; *p* < 0.05). Females were more likely to be aware of a celebrity who had a miscarriage than male students (16.9% versus 7.0%, Table 1). Students from Engineering and Food Science (*n* = 34; 18.3%) or Business and Commerce and Law (*n* = 14; 14.9%) disciplines were more likely to report that they did not know anyone with a miscarriage. Medicine and Health (*n* = 159; 74%), and Arts and Social Science (*n* = 130; 72.6%) were more likely to know someone who had a miscarriage (Table 1).

**Table 1:** University students’ characteristics by type of relationship with someone who had a miscarriage

		Do not know anyone, n (%)	Myself, partner, family or friend, n (%)	Celebrities, n (%)	*p*-value
Total*	674	85 (12.6)	489 (72.6)	100 (14.8)	
Sex					
Female	532	51 (9.6)	391 (73.5)	90 (16.9)	< 0.001
Male	142	34 (23.9)	98 (69.0)	10 (7.0)	
Age					
≤ 20	152	20 (13.2)	103 (67.8)	29 (19.1)	0.005
21–22	257	44 (17.1)	175 (68.1)	38 (14.8)	
≥ 23	265	21 (7.9)	211 (79.6)	33 (12.5)	
Marital status					
Single	554	78 (14.1)	394 (71.1)	82 (14.8)	0.045
Other (married, cohabiting…)	120	7 (5.8)	95 (79.2)	18 (15.0)	
Discipline					
Medicine and Health	215	21 (9.8)	159 (74.0)	35 (16.3)	0.023
Arts and Social Science	179	16 (8.9)	130 (72.6)	33 (18.4)	
Engineering & Food Science	186	34 (18.3)	135 (72.6)	17 (9.1)	
Business and Commerce & Law	94	14 (14.9)	65 (69.1)	15 (16.0)	
Level of study					
Undergraduate	535	76 (14.2)	378 (70.7)	81 (15.1)	0.035
Postgraduate	139	9 (6.5)	111 (79.9)	19 (13.7)	

^*^Missing data (*n* = 72)

Only 20% (*n* = 149) of students identified a mean rate of 20% for miscarriage. The remaining students underestimated or overestimated the rate of miscarriage (Table 2). Female students, older students and those who knew someone who had a miscarriage were more likely to identify the 20% rate of miscarriage. Students from Arts and Social Science (*n* = 45, 22.5%) and Medicine and Health (*n* = 52, 21.9) were more likely to estimate the correct rate of miscarriage (Table 2). A total of 96 (12.9%) students correctly responded that miscarriage happens up to 12 weeks of gestation (early miscarriage) or up to 24 weeks of gestation (late miscarriage). Overall, only 54 (6.2%) students were aware that miscarriage can happen from conception until 24 weeks of gestation. A quarter of all students (*n* = 179; 24%) thought miscarriage could happen at any stage of pregnancy.

**Table 2:** Grade of correct, underestimated and overestimated rate of miscarriage

Rate	Underestimated, *n* (%)	Correct (20%), *n* (%)	Overestimated, *n* (%)	*p*-value
Total	295 (39.9)	149 (20.1)	296 (40.0)	
Sex				
Female	192 (33.4)	125 (21.8)	257 (44.8)	< 0.001
Male	103 (62.0)	24 (14.5)	39 (23.5)	
Age				
≤ 20	66 (38.6)	32 (18.7)	73 (42.7)	0.803
21–22	118 (41.8)	54 (19.1)	110 (39.0)	
≥ 23	111 (38.7)	63 (22.0)	113 (39.4)	
Discipline				
Medicine and Health	92 (38.8)	52 (21.9)	93 (39.2)	0.207
Arts and Social Science	68 (34.0)	45 (22.5)	87 (43.5)	
Engineering & Food Science	95 (47.3)	33 (16.4)	73 (36.3)	
Business and Commerce & Law	40 (39.2)	19 (18.6)	43 (42.2)	
Known someone*				
Do not know anyone	50 (58.8)	9 (10.6)	26 (30.6)	< 0.001
Myself, partner, family or friends	181 (37.2)	114 (23.4)	192 (39.4)	
Celebrities	29 (29.0)	16 (16.0)	55 (55.0)	

*Missing data (*n* = 72)

The most common cause of miscarriage identified by the university students was chromosomal abnormalities in the baby, (*n* = 316; 42.4%), followed by medical conditions (*n* = 177; 23.7%) and lifestyles (*n* = 109; 14.6%). Chromosomal abnormalities of the baby were identified as the most common cause of miscarriage in a higher percentage of female students, older students (i.e. 23 years old or older), students who reported being married, divorced or cohabiting, students from Medicine and Health and for those students who knew a celebrity who had a miscarriage. Male students, younger and single students, students from Engineering and Food Science and Business and Commerce and Law, and students who reported that they did not know anyone who had a miscarriage were more likely to report lifestyles and the medical condition of the mother as the most common cause of miscarriage (Table 3).

**Table 3:** University Students’ awareness of most common cause of miscarriage

	Total	Lyfestyle, *n* (%)	Medical condition of mother, *n* (%)	Chromosomal abnormalities, *n* (%)	Psychological problems during pregnancy, *n* (%)	Incident during pregnancy, n (%)	Other, *n* (%)	*p*-value
Total	746	109 (14.6)	177 (23.7)	316 (42.4)	43 (5.8)	85 (11.4)	16 (2.1)	
Sex								
Female	577	78 (13.5)	127 (22.0)	251 (43.5)	34 (5.9)	73 (12.7)	14 (2.4)	0.060
Male	169	31 (18.3)	50 (29.6)	65 (38.5)	9 (5.3)	12 (7.1)	2 (1.2	
Age								
≤ 20	173	38 (22.0)	43 (24.9)	54 (31.2)	12 (6.9)	23 (13.3)	3 (1.7)	< 0.001
21–22	284	48 (16.9)	75 (26.4)	96 (33.8)	25 (8.8)	37 (13.0)	3 (1.1)	
≥ 23	289	23 (8.0)	59 (20.4)	166 (57.4)	6 (2.1)	25 (8.7)	10 (3.5)	
Marital status								
Single	617	103 (16.7)	152 (24.6)	232 (37.6)	38 (6.2)	79 (12.8)	13 (2.1)	< 0.001
Other (married, cohabiting…)	129	6 (4.7)	25 (19.4)	84 (65.1)	5 (3.9)	6 (4.7)	3 (2.3)	
Discipline								
Medicine and Health	238	29 (12.2)	33 (13.9)	143 (60.1)	10 (4.2)	20 (8.4)	3 (1.3)	< 0.001
Arts and Social Science	201	26 (12.9)	50 (24.9)	84 (41.8)	9 (4.5)	28 (13.9)	4 (2.0)	
Engineering & Food Science	203	32 (15.8)	63 (31.0)	64 (31.5)	17 (8.4)	22 (10.8)	5 (2.5)	
Business and Commerce & Law	104	22 (21.2)	31 (29.8)	25 (24.0)	7 (6.7)	15 (14.4)	4 (3.8)	
Known someone								
Do not know anyone	85	15 (17.6)	30 (35.3)	27 (31.8)	4 (4.7)	9 (10.6)	0 (0.0)	0.056
Myself, partner, family or friends	489	69 (14.1)	105 (21.5)	214 (43.8)	30 (6.1)	61 (12.5)	10 (2.0)	
Celebrities	100	12 (12.0)	19 (19.0)	52 (52.0)	3 (3.0)	10 (10.0)	4 (4.0)	
Rate of miscarriage								
Underestimate rate	295	42 (14.2)	89 (30.2)	107 (36.3)	19 (6.4)	34 (11.5)	4 (1.4)	0.022
Correct rate	149	22 (14.8)	30 (20.1)	72 (48.3)	3 (2.0)	18 (12.1)	4 (2.7)	
Over-estimate rate	296	44 (14.9)	54 (18.2)	136 (45.9)	21 (7.1)	33 (11.1)	8 (2.7)	

Students who correctly estimated the rate of miscarriage were more likely to select chromosomal abnormalities as the main cause of miscarriage (*n* = 72; 48.3% for correct rate of miscarriage, *n* = 136; 45.9% for overestimated rate and *n* = 107; 36.3% for underestimated rate; Table 3). Conversely, students who correctly identified the rate of miscarriage were less likely to select psychological problems as the main cause of miscarriage. Students who overestimated the rate of miscarriage were less likely to identify medical conditions of the mother as a cause of miscarriage, whereas those who underestimated were more likely to select it. Approximately 15% (underestimated rate *n* = 42; correct rate *n* = 22 and overestimated rate *n* = 44) of students selected lifestyle behaviour as the main cause of miscarriage independently of the selected rate of miscarriage (Table 3).

**Fig. 1:**
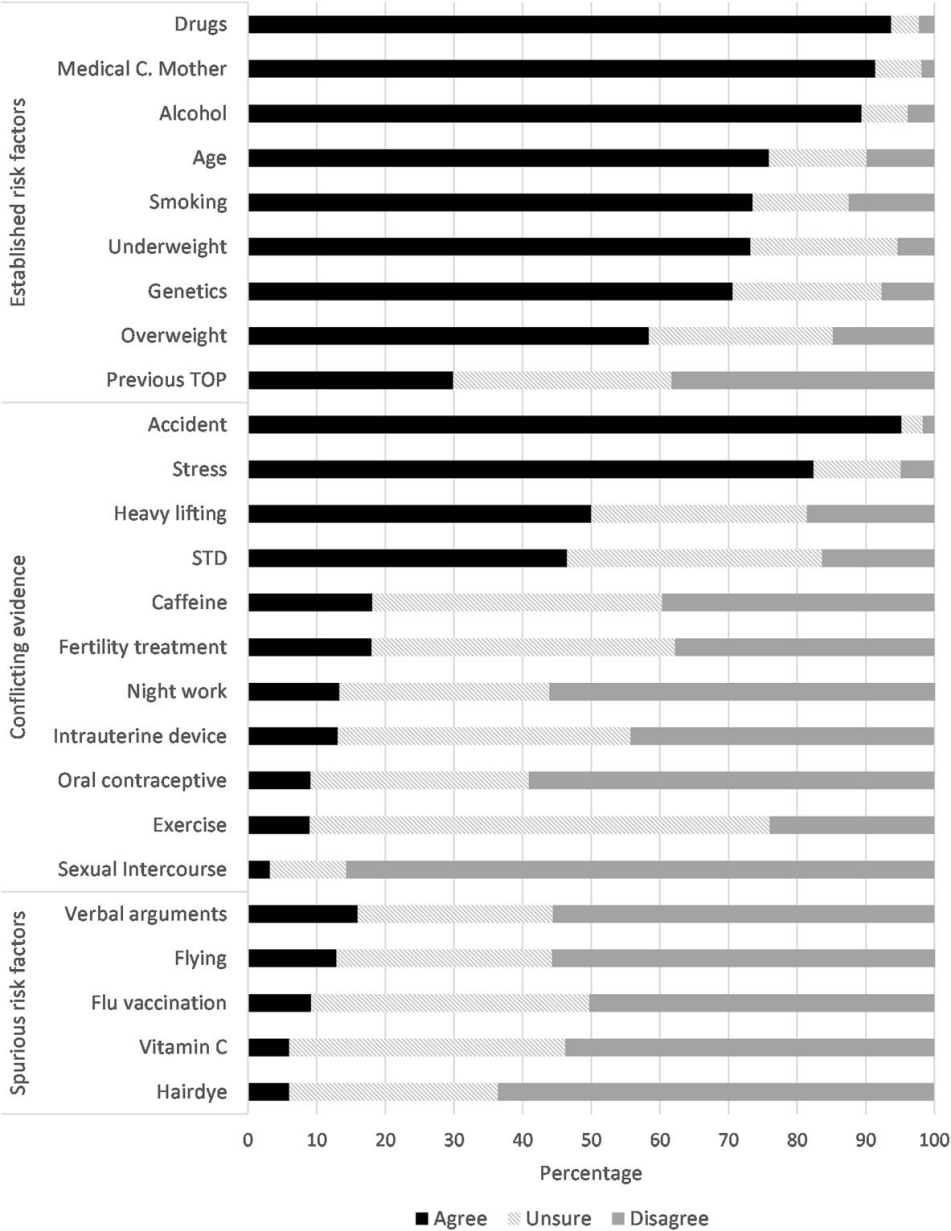
Percentage of most selected risk factors for miscarriage

The most reported risk factors for miscarriage were accident or fall, drugs, medical condition of the mother, alcohol, stress, age smoking and being underweight. Most students disagreed that sexual intercourse, hair dye, vitamin C and exercise were risk factors for miscarriage (Fig. 1).

Overall, the majority of college students correctly selected age (*n* = 566; 88%) and medical conditions of the mother (*n* = 682; 98%) as contributory risk factors for miscarriage. No statistically significant differences between agree or disagree responses for age or for medical conditions of mother were found between groups (Additional file 1: Table S1). However, students from Arts and Social Science were more likely to be unsure about age as a risk factor (aOR 2.78; 95% CI 1.52–5.09). Students of 21 years of age or older were more likely to identify chromosomal abnormalities as a causative factor for miscarriage than those aged 20 years old or younger (students aged 21–22: aOR 0.27; 95% CI 0.12–0.61 and students aged 23 years old or older: aOR 0.48; 95% CI 0.24–0.96; Additional file 1: Table S1). Students from Arts and Social Science or Business and Commerce and Law more frequently did not identify chromosomal abnormalities as a potential causative factor compared to college students from Medical and Health (aOR 2.40; 95% CI 1.01–5.73 and aOR 3.0; 95% CI 1.16–7.73 respectively; Additional file 1: Table S1).

Male students were more likely to agree that smoking was a risk factor for miscarriage compared to female students (aOR 0.47; 95% CI 0.24–0.94). Older students (i.e. 23 years old or older) disagreed more frequently that smoking was a risk factor for miscarriage compared to students who were 20 years old or younger (aOR 2.09; 95% CI 1.08–4.07). Compared to students from Medicine and Health, the remaining disciplines disagreed more frequently that smoking was a risk factor. For alcohol, older students and those from Business and Commerce and Law were more likely to disagree that it was a risk factor for miscarriage (Additional file 1: Table S1).

Students from Arts and Social Science were more likely to identify flu vaccination as a risk factor for miscarriage (*n* = 25; 26.9%; Additional file 1: Table S2). Students from Engineering and Food Science and Business and Commerce and Law were more likely to identify verbal arguments as a risk factor for miscarriage (aOR 0.56; 95% CI 0.31–0.99 and aOR 0.42; 95% CI 0.21–0.82). Students between 21 and 22 years old were more likely to be unsure that vitamin C was a risk factor for miscarriage compared to younger students (aOR 2.85; 95% CI 1.21–6.72; Additional file 1: Table S2). Only students who were 23 years old or older were more likely to identify vitamin C as a spurious risk factor compared to students who were 20 years old or younger (aOR 2.34; 95% CI 1.03–5.34; Additional file 1: Table S2).

Among the remaining potential causative risk factors for miscarriage, male students were less likely to identify working night shifts and previous termination of pregnancy (TOP) as risk factors (aOR 0.45; 95% CI 0.25–0.80and aOR 0.44; 95% CI 0.26–0.72). Older students (i.e. 23 years old or older) were less likely to identify caffeine as a risk factor (aOR 2.61; 95%CI 1.45–4.70). Compared to students from the college of Medicine and Health, those from Business and Commerce and Law were less likely to identify sexually transmitted disease, previous TOP and being underweight as contributory risk factors for miscarriage (aOR 3.39; 95% CI 1.77–6.51 and aOR 2.20; 95% CI 1.13–4.25 and aOR 2.79; 95% CI 1.10–7.03). Students from Engineering and Food Science were less likely to identify night work as a risk factor, but were more likely to consider stress as a contributory risk factor for miscarriage compared to Medicine and Health students (aOR 2.06; 95%CI 1.08–3.93 and aOR 0.36; 95% CI 0.13–0.98). The odds of not identifying oral contraceptive as a cause of miscarriage were lower for students who overestimated the rate of miscarriage compared to those who correctly identified the rate (OR: 0.30; 95% CI 0.12–0.75). Finally, only students from Arts and Social Science were more likely to identify heavy lifting as a risk factor.

## Discussion

### Main findings

This cross-sectional study provides insight into university students’ awareness of prevalence and risk factors of miscarriage. The findings of this study illustrate that common misunderstandings still prevail regarding the aetiology of miscarriage, suggesting a deficiency in formal information and access to information related to reproductive health. For example, only 20% of the students correctly identified the prevalence of miscarriage at 20%, and almost 30% incorrectly believed the prevalence of miscarriage is less common than 10%. Female students were more likely to identify the correct rate, but also to overestimate it, and male students tended to underestimate it. Almost one-quarter of the students believed miscarriage can happen from conception until birth, and 87% of the students erroneously selected the weeks of gestation at which miscarriage occurs. Females students, older students, those from Medicine and Health, those who were aware of a celebrity who had a miscarriage, and those who identified the correct rate of miscarriage were more likely to identify chromosomal abnormalities as the most common cause of miscarriage. However, this was only identified by 43% of the total sample.

### Strengths and limitations

The nature of the study design implies that data were collected at one point in time. Previous studies have found an association between ethnicity and religion and the perception of risk factors for miscarriage [25], however we did not include this information in our survey and no comparison can be made. One of the main limitations is that a higher percentage of female students responded to the survey compared to male students. Although similar gender distributions were reported at UCC in the academic year 2006/2007 (36% male and 64% females) [26], recent overall data shows a more equal gender distribution for third-level graduates in the Republic of Ireland in 2016, with 52.2% of the students being female [27]. This percentage is similar to the European Union (EU-28) in 2015 [28]. Nevertheless, our sample seems to be representative of the overall distribution of male and females by discipline. In 2016, women represented more than three out of four (76.4%) graduates in Health, and more than four out of five (82.4%) graduates in Engineering were male [27] in the Republic of Ireland.

No standardised instrument of relevance was found in the literature for the purpose of this study; and therefore our survey was not validated. A multidisciplinary team specialised in pregnancy loss developed and reviewed all questions. In addition, a patient advocate for women who experience pregnancy loss also reviewed the questionnaire to ensure clarity. To our knowledge, this is one of the largest studies exploring the knowledge of rates and risk factors for miscarriage among college students from multiple disciplines, representing the main strength of this study.

### Comparison with other studies

Our study is in keeping with the results of two previous studies [25, 29]. In a cross-sectional study including 1084 adults located in 49 states within the United States, Bardos et al. found that half of the participants believed that miscarriage was uncommon, occurring in 5% or less of all pregnancies. Similar to our results, it also found that approximately one fifth of the respondents incorrectly believed that lifestyle behaviours such as consumption of drugs, alcohol or tobacco were the only cause of miscarriage. In addition, men were more likely to identify lifestyle behaviours as a contributing risk factor for miscarriage. Also, participants with a higher educational degree identified chromosomal abnormalities more frequently as a cause of miscarriage compare to less educated respondents [25]. It is important to note that approximately 80% of these participants attended some college or medical school. Interestingly, in our study, male students were also more likely to identify smoking as a contributing risk factor. In another study, Delgado et at assessed awareness among undergraduate students related to preconception health and pregnancy. Results showed a low to moderate level of awareness, with women having a slightly higher awareness than men [29].

Assessing the reasons behind overestimating or underestimating the risk of miscarriage is difficult to understand [30]. It could be possible that students who overestimate the risk of miscarriage were under unnecessary stress or anxiety at the time of this study. Some studies have shown a link between psychological distress and anticipatory representations of possible future threats or overestimating the risk of a disease [31, 32]. No studies have evaluated college students’ psychological and lifestyles factors and perception of risk of pregnancy loss; therefore, more research needs to be done to assess which are the underlying factors that might impact on population’s perception of risk of pregnancy loss.

### Implications

Despite the high occurrence of miscarriage, some studies highlight the potential barriers that might influence the lack of awareness of this topic among the general public. For example, the existence of guilt, shame or feeling responsible for the pregnancy loss might have reinforced the reclusion of the topic exclusively to the close family or friends, or in some cases, only among the couple who experience miscarriage [33, 34]. This has led to miscarriage being a “taboo” or “unspoken” topic in some cultures, increasing the chance of the causes of miscarriage being surrounded by myths and folklore [25, 35]. The potential benefits of promoting healthy behaviours, lifestyle, mental and social factors during women and men’s reproductive years has been increasingly accepted in the medical and scientific community [13, 36].In this context, preconception health care is a unique opportunity to increase personal responsibility and awareness of risk factors and adverse pregnancy outcomes during the reproductive years of this targeted population [16].

Universities are underused settings for improving preconception health among the community. They provide an opportunity to reach a population with a diverse socioeconomic and gender background. In a scoping review of 29 preconception health care interventions evaluations, six of them were delivered at a School, college or university settings [17]. All of them reported an improvement in preconception health knowledge [29, 37–40]; however, most of the interventions were provided to women who were identified as being at-risk of developing adverse maternal outcomes, and men were not generally included in the interventions [37]. Although the Republic of Ireland has one of the highest birth rates in Europe [41], to our knowledge, there are no preconception healthcare intervention programmes or clinical practice guidelines focused on improved preconception healthcare in higher education settings.

## Conclusion

According to our results and the little evidence available, misunderstanding of causes and risk factors for miscarriage is a public health issue. The findings of this study highlight an opportunity for public health interventions to improve reproductive health education. Universally preconception healthcare programmes successfully provide health promotion strategies to increase awareness of potential adverse outcomes in pregnancy. In particular, University settings are an ideal opportunity to reach a targeted population.

## Additional file

Additional file 1: **Table S1.** Odds Ratios of agreement with strong risk factors for miscarriage. **Table S2.** Odds Ratios of disagreement with spurious risk factors for miscarriage. (DOCX 33 kb)

## Abbreviations

aOR: Adjusted odds ratios
CI: Confidence intervals
EU-28: European Union of 28 member states
OR: Unadjusted odds ratios
SD: Standard deviation
TOP: Termination of pregnancy
UCC: University College Cork
