# Supplementary material for: University students’ awareness of causes and risk factors of miscarriage: a cross-sectional study
Source: BMC Womens Health. 2018 Nov 19;18:188. doi: 10.1186/s12905-018-0682-1 (PMC6245715; doi:10.1186/s12905-018-0682-1)
Supplement: Supplementary file 1 — Table S1. Odds Ratios of agreement with strong risk factors for miscarriage. Table S2. Odds Ratios of disagreement with spurious risk factors for miscarriage. (DOCX 33 kb) [file 12905_2018_682_MOESM1_ESM.docx]

**Supplementary tables**

**Table S1. Odds Ratios of agreement with strong risk factors for miscarriage.**

| **Age** | **Agree**  **n (%)** | **Disagree**  **n (%)** | **OR (95%CI)** | **p-value** | **aOR (95%CI)** | **p-value** |
| --- | --- | --- | --- | --- | --- | --- |
| **Total** | 566 (88.4) | 74 (11.6) |  |  |  |  |
| **Sex** |  |  |  |  |  |  |
| Female | 435 (88.4) | 57 (11.6) | 1 | (ref.) | 1 | (ref.) |
| Male | 131 (88.5) | 17 (11.5) | 0.99 (0.56-1.8) | 0.97 | 0.84 (0.42-1.71) | 0.84 |
| **Age** |  |  |  |  |  |  |
| ≤20 | 131 (87.3) | 19 (12.7) | 1 | (ref.) | 1 | (ref.) |
| 21-22 | 211 (89.4) | 25 (10.6) | 0.82 (0.43-1.54) | 0.53 | 1.14 (0.55-2.34) | 0.73 |
| ≥23 | 224 (88.2) | 30 (11.8) | 0.92 (0.50-1.71) | 0.80 | 1.34 (0.65-2.74) | 0.42 |
| **Discipline** |  |  |  |  |  |  |
| Medicine and Health | 190 (88.0) | 26 (12.0) | 1 | (ref.) | 1 | (ref.) |
| Arts and Social Science | 140 (88.1) | 19 (11.9) | 0.99 (0.53-1.86) | 0.98 | 1.10 (0.56-2.16) | 0.77 |
| Engineering & Food Science | 165 (93.2) | 12 (6.8) | 0.53 (0.26-1.09) | 0.08 | 0.46 (0.21-1.10) | 0.07 |
| Business and Commerce & Law | 71 (80.7) | 17 (19.3) | 1.75 (0.90-3.42) | 0.10 | 1.96 (0.95-4.03) | 0.07 |
| **Known someone** | 515 (88.6) | 66 (11.4) |  |  |  |  |
| Do not know anyone | 68 (88.3) | 9 (11.7) | 1 | (ref.) | 1 | (ref.) |
| Myself, partner, family or friends | 378 (90.4) | 40 (9.6) | 0.80 (0.37-1.72) | 0.57 | 0.74 (0.33-1.65) | 0.46 |
| Celebrities | 69 (80.2) | 17 (19.8) | 1.86 (0.78-4.47) | 0.16 | 1.60 (0.63-4.03) | 0.32 |
| **Rate of miscarriage** |  |  |  |  |  |  |
| Correct rate | 118 (92.9) | 9 (7.1) | 1 | (ref.) | 1 | (ref.) |
| Over-estimate fertility | 221 (86.3) | 35 (13.7) | 2.08 (0.97-4.47) | 0.06 | 1.98 (0.90-4.34) | 0.09 |
| Underestimate miscarriage | 224 (88.5) | 29 (11.5) | 1.70 (0.78-3.70) | 0.18 | 1.65 (0.72-3.78) | 0.23 |
| **Chromosomal abnormalities** | **Agree**  **n (%)** | **Disagree**  **n (%)** | **OR (95%CI)** | **p-value** | **aOR (95%CI)** | **p-value** |
| **Total** | 527 (90.2) | 57 (9.8) |  |  |  |  |
| **Sex** |  |  |  |  |  |  |
| Female | 404 (90.4) | 43 (9.6) | 1 | (ref.) | 1 | (ref.) |
| Male | 123 (89.8) | 14 (10.2) | 1.07 (0.57-2.02) | 0.84 | 1.20 (0.55-2.61) | 0.65 |
| **Age** |  |  |  |  |  |  |
| ≤20 | 110 (81.5) | 25 (18.5) | 1 | (ref.) | 1 | (ref.) |
| 21-22 | 202 (94.8) | 11 (5.2) | **0.24 (0.11-0.51)** | **0.00** | **0.27 (0.12-0.61)** | **0.00** |
| ≥23 | 215 (91.1) | 21 (8.9) | **0.43 (0.23-0.80)** | **0.01** | **0.48 (0.24-0.96)** | **0.04** |
| **Discipline** |  |  |  |  |  |  |
| Medicine and Health | 187 (94.9) | 10 (5.1) | 1 | (ref.) | 1 | (ref.) |
| Arts and Social Science | 126 (86.3) | 20 (13.7) | **2.97 (1.34-6.55)** | **0.01** | **2.40 (1.01-5.73)** | **0.05** |
| Engineering & Food Science | 144 (91.1) | 14 (8.9) | 1.82 (0.79-4.21) | 0.16 | 1.53 (0.61-3.87) | 0.37 |
| Business and Commerce & Law | 70 (84.3) | 13 (15.7) | **3.47 (1.46-8.28)** | **0.01** | **3.0 (1.16-7.73)** | **0.02** |
| **Known someone** | 474 (90.6) | 49 (9.4) |  |  |  |  |
| Do not know anyone | 56 (88.9) | 7 (11.1) | 1 | (ref.) | 1 | (ref.) |
| Myself, partner, family or friends | 346 (91.1) | 34 (8.9) | 0.80 (0.33-1.86) | 0.58 | 0.67 (0.27-1.71) | 0.40 |
| Celebrities | 72 (90.0) | 8 (10.0) | 0.90 (030-2.60) | 0.83 | 0.70 (0.21-2.37) | 0.60 |
| **Rate of miscarriage** |  |  |  |  |  |  |
| Correct rate | 105 (89.7) | 12 (10.3) | 1 | (ref.) | 1 | (ref.) |
| Over-estimate fertility | 212 (90.6) | 22 (9.4) | 0.91 (0.43-1.91) | 0.80 | 0.89 (0.40-1.97) | 0.77 |
| Underestimate miscarriage | 207 (90.4) | 22 (9.6) | 0.93 (0.44-1.95) | 0.85 | 0.76 (0.33-1.79) | 0.53 |
| **Smoking** | **Agree**  **n (%)** | **Disagree**  **n (%)** | **OR (95%CI)** | **p-value** | **aOR (95%CI)** | **p-value** |
| **Total** | 548 (85.5) | 93 (14.5) |  |  |  |  |
| **Sex** |  |  |  |  |  |  |
| Female | 410 (83.8) | 79 (16.2) | 1 | (ref.) | 1 | (ref.) |
| Male | 138 (90.8) | 14 (9.2) | **0.53 (0.29-0.96)** | **0.04** | **0.47 (0.24-0.94)** | **0.03** |
| **Age** |  |  |  |  |  |  |
| ≤20 | 131 (87.9) | 18 (12.1) | 1 | (ref.) | 1 | (ref.) |
| 21-22 | 211 (85.8) | 35 (14.2) | 1.21 (0.66-2.22) | 0.54 | 1.66 (0.86-3.22) | 0.13 |
| ≥23 | 206 (83.7) | 40 (16.3) | 1.41 (0.78-2.57) | 0.26 | **2.09 (1.08-4.07)** | **0.03** |
| **Discipline** |  |  |  |  |  |  |
| Medicine and Health | 197 (92.9) | 15 (7.1) | 1 | (ref.) | 1 | (ref.) |
| Arts and Social Science | 139 (79.9) | 35 (20.1) | **3.31 (1.74-6.29)** | **0.00** | **3.53 (1.79-6.97)** | **0.00** |
| Engineering & Food Science | 137 (81.5) | 31 (18.5) | **2.97 (1.55-5.72)** | **0.00** | **3.46 (1.72-6.94)** | **0.00** |
| Business and Commerce & Law | 75 (86.2) | 12 (13.8) | 2.10 (0.94-4.70) | 0.07 | **2.44 (1.04-5.74)** | **0.04** |
| **Known someone** | 491 (85.1) | 86 (14.9) |  |  |  |  |
| Do not know anyone | 63 (87.5) | 9 (12.5) | 1 | (ref.) | 1 | (ref.) |
| Myself, partner, family or friends | 355 (84.7) | 64 (15.3) | 1.26 (0.60-2.67) | 0.54 | 1.15 (0.53-2.51) | 0.73 |
| Celebrities | 73 (84.9) | 13 (15.1) | 1.25 (0.50-3.11) | 0.64 | 1.16 (0.44-3.03) | 0.77 |
| **Rate of miscarriage** |  |  |  |  |  |  |
| Correct rate | 104 (85.2) | 18 (14.8) | 1 | (ref.) | 1 | (ref.) |
| Over-estimate fertility | 219 (94.9) | 39 (15.1) | 1.03 (0.56-1.89) | 0.93 | 1.03 (0.54-1.96) | 0.93 |
| Underestimate miscarriage | 220 (85.9) | 36 (14.1) | 0.95 (0.51-1.74) | 0.86 | 1.05 (0.54-2.06) | 0.88 |
| **Alcohol** | **Agree**  **n (%)** | **Disagree**  **n (%)** | **OR (95%CI)** | **p-value** | **aOR (95%CI)** | **p-value** |
| **Total** | 667 (95.8) | 29 (4.2) |  |  |  |  |
| **Sex** |  |  |  |  |  |  |
| Female | 510 (95.3) | 25 (4.7) | 1 | (ref.) | 1 | (ref.) |
| Male | 157 (97.5) | 4 (2.5) | 0.52 (0.18-1.52) | 0.23 | 0.54 (0.17-1.68) | 0.29 |
| **Age** |  |  |  |  |  |  |
| ≤20 | 161 (98.2) | 3 (1.8) | 1 | (ref.) | 1 | (ref.) |
| 21-22 | 259 (96.6) | 9 (3.4) | 1.87 (0.50-7.00) | 0.36 | 1.39 (0.35-5.56) | 0.64 |
| ≥23 | 247 (93.6) | 17 (6.4) | **3.70 (1.07-12.81)** | **0.04** | **3.74 (1.03-13.64)** | **0.05** |
| **Discipline** |  |  |  |  |  |  |
| Medicine and Health | 216 (95.6) | 10 (4.4) | 1 | (ref.) | 1 | (ref.) |
| Arts and Social Science | 183 (96.3) | 7 (3.7) | 0.83 (0.31-2.21) | 0.70 | 0.89 (0.28-2.84) | 0.85 |
| Engineering & Food Science | 185 (97.4) | 5 (2.6) | 0.58 (0.20-1.74) | 0.33 | 0.95 (0.30-3.06) | 0.93 |
| Business and Commerce & Law | 83 (92.2) | 7 (7.8) | 1.82 (0.67-4.94) | 0.24 | **3.02 (1.01-9.01)** | **0.05** |
| **Known someone** | 603 96.0) | 25 (4.0) |  |  |  |  |
| Do not know anyone | 75 (93.8) | 5 (6.3) | 1 | (ref.) | 1 | (ref.) |
| Myself, partner, family or friends | 442 (96.5) | 16 (3.5) | 0.54 (0.19-1.53) | 0.25 | 0.43 (0.15-1.29) | 0.13 |
| Celebrities | 86 (95.6) | 4 (4.4) | 0.70 (0.18-2.69) | 0.60 | 0.55 (0.13-2.27) | 0.40 |
| **Rate of miscarriage** |  |  |  |  |  |  |
| Correct rate | 136 (95.1) | 7 (4.9) | 1 | (ref.) | 1 | (ref.) |
| Over-estimate fertility | 261 (96.0) | 11 (4.0) | 0.82 (0.31-2.16) | 0.69 | 0.69 (0.24-1.95) | 0.48 |
| Underestimate miscarriage | 265 (96.0) | 11 (4.0) | 0.81 (0.31-2.13) | 0.66 | 0.75 (0.26-2.16) | 0.60 |
| **Medical condition of mother** | **Agree**  **n (%)** | **Disagree**  **n (%)** | **OR (95%CI)** | **p-value** | **aOR (95%CI)** | **p-value** |
| **Total** | 682 (98.0) | 14 (2.0) |  |  |  |  |
| **Sex** |  |  |  |  |  |  |
| Female | 529 (98.1) | 10 (1.9) | 1 | (ref.) | 1 | (ref.) |
| Male | 153 (97.5) | 4 (2.5) | 1.38 (0.43-4.47) | 0.59 | 0.66 (0.16-2.72) | 0.57 |
| **Age** |  |  |  |  |  |  |
| ≤20 | 152 (97.4) | 4 (2.6) | 1 | (ref.) | 1 | (ref.) |
| 21-22 | 265 (99.3) | 2 (0.7) | 0.29 (0.05-1.58) | 0.15 | 0.29 (0.05-1.66) | 0.17 |
| ≥23 | 265 )97.1) | 8 (2.9) | 1.15 (0.34-3.87) | 0.83 | 1.27 (0.34-4.73) | 0.72 |
| **Discipline** |  |  |  |  |  |  |
| Medicine and Health | 225 (98.7) | 3 (1.3) | 1 | (ref.) | 1 | (ref.) |
| Arts and Social Science | 179 (97.3) | 5 (2.7) | 2.10 (0.49-8.89) | 0.32 | 1.74 (0.37-8.14) | 0.49 |
| Engineering & Food Science | 185 (97.9) | 4 (2.1) | 1.62 (0.36-7.34) | 0.53 | 1.76 (0.35-8.81) | 0.49 |
| Business and Commerce & Law | 93 (97.9) | 2 (2.1) | 1.61 (0.27-9.81) | 0.60 | 1.79 (0.28-11.62) | 0.54 |
| **Known someone** | 618 (97.9) | 13 (2.1) |  |  |  |  |
| Do not know anyone | 78 (96.3) | 3 (3.7) | 1 | (ref.) | 1 | (ref.) |
| Myself, partner, family or friends | 448 (98.5) | 7 (1.5) | 0.41 (0.10-1.61) | 0.20 | 0.42 (0.10-1.82) | 0.25 |
| Celebrities | 92 (96.8) | 3 (3.2) | 0.85 (1.67-4.32) | 0.84 | 1.04 (0.18-6.05) | 0.69 |
| **Rate of miscarriage** |  |  |  |  |  |  |
| Correct rate | 139 (98.6) | 2 (1.4) | 1 | (ref.) | 1 | (ref.) |
| Over-estimate fertility | 272 (99.3) | 2 (0.7) | 0.51 (0.07-3.67) | 0.50 | 0.45 (0.06-3.27) | 0.43 |
| Underestimate miscarriage | 268 (96.4) | 10 (3.6) | 2.59 (0.56-12.0) | 0.22 | 2.36 (0.48-11.62) | 0.29 |

**Table S2. Odds Ratios of disagreement with spurious risk factors for miscarriage.**

| **Flu vaccine** | **Agree**  **n (%)** | **Disagree**  **n (%)** | **OR (95%CI)** | **p-value** | **aOR (95%CI)** | **p-value** |
| --- | --- | --- | --- | --- | --- | --- |
| **Total** | 69 (15.5) | 375 (85.4) |  |  |  |  |
| **Sex** |  |  |  |  |  |  |
| Female | 57 (17.0) | 278 (83.0) | 1 | (ref.) | 1 | (ref.) |
| Male | 12 (11.0) | 97 (89.0) | 1.66 (0.85-3.22) | 0.14 | **2.41 (1.00 -5.78)** | **0.05** |
| **Age** |  |  |  |  |  |  |
| ≤20 | 17 (17.7) | 79 (82.3) | 1 | (ref.) | 1 | (ref.) |
| 21-22 | 29 (18.0) | 132 (82.0) | 0.98 (0.51-1.90) | 0.95 | 0.75 (0.35-1.59) | 0.45 |
| ≥23 | 23 (12.3) | 164 (87.7) | 1.53 (0.78-3.04) | 0.22 | 1.03 (0.48-2.24) | 0.93 |
| **Discipline** |  |  |  |  |  |  |
| Medicine and Health | 17 (9.9) | 155 (90.1) | 1 | (ref.) | 1 | (ref.) |
| Arts and Social Science | 25 (26.9) | 68 (73.1) | **0.30 (0.15-0.59)** | **0.00** | **0.31 (0.15-0.64)** | **0.00** |
| Engineering & Food Science | 14 (11.8) | 105 (88.2) | 0.82 (0.34-1.74) | 0.61 | 0.79 (0.36-1.75) | 0.56 |
| Business and Commerce & Law | 13 (21.7) | 47 (78.3) | **0.40 (0.18-0.88)** | **0.02** | 0.49 (0.20-1.22) | 0.13 |
| **Known someone** | 60 (14.7) | 347 (85.3) |  |  |  |  |
| Do not know anyone | 4 (7.3) | 51 (92.7) | 1 | (ref.) | 1 | (ref.) |
| Myself, partner, family or friends | 46 (15.7) | 247 (84.3) | 0.42 (0.15-1.22) | 0.11 | 0.41 (0.14-1.22) | 0.11 |
| Celebrities | 10 (16.9) | 49 (83.1) | 0.38 (0.11-1.31) | 0.13 | 0.46 (0.13-1.62) | 0.23 |
| **Rate of miscarriage** |  |  |  |  |  |  |
| Correct rate | 13 (13.1) | 86 (86.9) | 1 | (ref.) | 1 | (ref.) |
| Over-estimate fertility | 26 (15.8) | 139 (84.2) | 0.81 (0.39-1.66) | 0.56 | 0.79 (0.37-1.70) | 0.55 |
| Underestimate miscarriage | 28 (15.8) | 149 (84.2) | 0.80 (0.40-1.64) | 0.55 | 0.61 (0.28-1.35) | 0.22 |
| **Flying** | **Agree**  **n (%)** | **Disagree**  **n (%)** | **OR (95%CI)** | **p-value** | **aOR (95%CI)** | **p-value** |
| **Total** | 96 (18.8) | 416 (81.3) |  |  |  |  |
| **Sex** |  |  |  |  |  |  |
| Female | 80 (20.5) | 310 (76.5) | 1 | (ref.) | 1 | (ref.) |
| Male | 16 (13.1) | 106 (86.9) | 1.71 (0.96-3.05) | 0.07 | 1.65 (0.83-3.26) | 0.15 |
| **Age** |  |  |  |  |  |  |
| ≤20 | 22 (20.0) | 88 (80.0) | 1 | (ref.) | 1 | (ref.) |
| 21-22 | 36 (18.7) | 157 (81.3) | 1.10 (0.60-1.97) | 0.77 | 1.10 (0.58-2.09) | 0.77 |
| ≥23 | 38 (18.2) | 171 (81.8) | 1.13 (0.63-2.02) | 0.69 | 1.13 (0.59-2.18) | 0.71 |
| **Discipline** |  |  |  |  |  |  |
| Medicine and Health | 33 (18.6) | 144 (81.4) | 1 | (ref.) | 1 | (ref.) |
| Arts and Social Science | 26 (20.3) | 102 (79.7) | 0.90 (0.51-1.60) | 0.72 | 0.94 (0.51-1.73) | 0.84 |
| Engineering & Food Science | 21 (15.3) | 116 (84.7) | 1.27 (0.70-2.31) | 0.44 | 1.28 (0.66-2.46) | 0.46 |
| Business and Commerce & Law | 16 (22.9) | 54 (77.1) | 0.77 (0.39-1.52) | 0.46 | 0.96 (0.45-2.06) | 0.92 |
| **Known someone** | 83 (17.8) | 383 (82.2) |  |  |  |  |
| Do not know anyone | 7 (10.9) | 57 (89.1) | 1 | (ref.) | 1 | (ref.) |
| Myself, partner, family or friends | 62 (18.6) | 272 (81.4) | 0.54 (0.23-1.24) | 0.15 | 0.62 (0.26-1.45) | 0.27 |
| Celebrities | 14 (20.6) | 54 (79.4) | 0.47 (0.18-1.26) | 0.14 | 0.60 (0.22-1.64) | 0.32 |
| **Rate of miscarriage** |  |  |  |  |  |  |
| Correct rate | 20 (20.6) | 77 (79.4) | 1 | (ref.) | 1 | (ref.) |
| Over-estimate fertility | 40 (20.2) | 158 (79.8) | 1.03 (0.56-1.87) | 0.93 | 1.03 (0.54-1.94) | 0.94 |
| Underestimate miscarriage | 35 (16.4) | 179 (83.6) | 1.33 (0.72-2.45) | 0.36 | 1.20 (0.62-2.33) | 0.60 |
| **Hair dye** | **Agree**  **n (%)** | **Disagree**  **n (%)** | **OR (95%CI)** | **p-value** | **aOR (95%CI)** | **p-value** |
| **Total** | 45 (8.7) | 474 (91.3) |  |  |  |  |
| **Sex** |  |  |  |  |  |  |
| Female | 38 (9.5) | 363 (90.5) | 1 | (ref.) | 1 | (ref.) |
| Male | 7 (5.9) | 111 (94.1) | 1.67 (0.72-3.82) | 0.23 | 1.87 (0.61-5.76) | 0.27 |
| **Age** |  |  |  |  |  |  |
| ≤20 | 10 (8.4) | 109 (91.6) | 1 | (ref.) | 1 | (ref.) |
| 21-22 | 23 (11.6) | 175 (88.4) | 0.70 (0.32-1.52) | 0.37 | 0.80 (0.34-1.90) | 0.62 |
| ≥23 | 12 (5.9) | 190 (94.1) | 1.45 (0.61-3.47) | 0.40 | 2.18 (0.79-6.04) | 0.14 |
| **Discipline** |  |  |  |  |  |  |
| Medicine and Health | 14 (8.2) | 156 (91.8) | 1 | (ref.) | 1 | (ref.) |
| Arts and Social Science | 17 (12.3) | 121 (87.7) | 0.64 (0.30-1.35) | 0.24 | 0.85 (0.35-2.02) | 0.70 |
| Engineering & Food Science | 8 (5.8) | 131 (94.2) | 1.47 (0.60-3.61) | 0.40 | 1.77 (0.65-4.78) | 0.26 |
| Business and Commerce & Law | 6 (8.3) | 66 (91.7) | 0.99 (0.36-2.68) | 0.98 | 1.51 (0.45-5.07) | 0.51 |
| **Known someone** | 35 (7.4) | 441 (92.6) |  |  |  |  |
| Do not know anyone | 5 (7.1) | 65 (92.9) | 1 | (ref.) | 1 | (ref.) |
| Myself, partner, family or friends | 29 (8.7) | 306 (91.3) | 0.81 (0.30-2.18) | 0.68 | 0.90 (0.32-2.54) | 0.83 |
| Celebrities | 1 (1.4) | 70 (98.6) | 5.39 (0.61-47.32) | 0.13 | 7.32 (0.80-66.86) | 0.08 |
| **Rate of miscarriage** |  |  |  |  |  |  |
| Correct rate | 7 (6.4) | 102 (93.6) | 1 | (ref.) | 1 | (ref.) |
| Over-estimate fertility | 24 (11.7) | 181 (88.3) | 0.52 (0.22-1.24) | 0.14 | 0.68 (0.27-1.73) | 0.42 |
| Underestimate miscarriage | 14 (6.9) | 189 (93.1) | 0.93 (0.36-2.37) | 0.87 | 0.97 (0.35-2.69) | 0.95 |
| **Verbal arguments** | **Agree**  **n (%)** | **Disagree**  **n (%)** | **OR (95%CI)** | **p-value** | **aOR (95%CI)** | **p-value** |
| **Total** | 119 (22.3) | 415 (77.7) |  |  |  |  |
| **Sex** |  |  |  |  |  |  |
| Female | 95 (22.6) | 325 (77.4) | 1 | (ref.) | 1 | (ref.) |
| Male | 24 (21.1) | 90 (78.9) | 1.10 (0.66-1.82) | 0.72 | 0.96 (0.54-1.72) | 0.90 |
| **Age** |  |  |  |  |  |  |
| ≤20 | 26 (22.2) | 91 (77.8) | 1 | (ref.) | 1 | (ref.) |
| 21-22 | 54 (26.9) | 147 (73.1) | 0.78 (0.46-1.33) | 0.36 | 0.65 (0.36-1.19) | 0.17 |
| ≥23 | 39 (18.1) | 177 (81.9) | 1.30 (0.74-2.26) | 0.36 | 1.03 (0.55-1.94) | 0.93 |
| **Discipline** |  |  |  |  |  |  |
| Medicine and Health | 30 (16.3) | 154 (83.7) | 1 | (ref.) | 1 | (ref.) |
| Arts and Social Science | 28 (21.5) | 102 (78.5) | 0.71 (0.40-1.26) | 0.24 | 0.85 (0.45-1.60) | 0.61 |
| Engineering & Food Science | 38 (25.9) | 109 (74.1) | **0.56 (0.33-0.96)** | **0.03** | **0.56 (0.31-0.99)** | **0.05** |
| Business and Commerce & Law | 23 (31.5) | 50 (68.5) | **0.42 (0.23-0.80)** | **0.01** | **0.42 (0.21-0.82)** | **0.01** |
| **Known someone** | 105 (21.6) | 382 (78.4) |  |  |  |  |
| Do not know anyone | 11 (19.0) | 47 (81.0) | 1 | (ref.) | 1 | (ref.) |
| Myself, partner, family or friends | 75 (20.9) | 284 (79.1) | 0.89 (0.44-1.79) | 0.74 | 0.69 (0.33-1.46) | 0.33 |
| Celebrities | 19 (27.1) | 51 (72.9) | 0.63 (0.27-1.46) | 0.28 | 0.51 (0.21-1.24) | 0.14 |
| **Rate of miscarriage** |  |  |  |  |  |  |
| Correct rate | 17 (16.8) | 84 (83.2) | 1 | (ref.) | 1 | (ref.) |
| Over-estimate fertility | 54 (24.4) | 167 (75.6) | 0.63 (0.34-1.15) | 0.13 | 0.71 (0.37-1.34) | 0.29 |
| Underestimate miscarriage | 48 (22.7) | 163 (77.3) | 0.69 (0.37-1.27) | 0.23 | 0.75 (0.39-1.45) | 0.39 |
| **Vitamin C** | **Agree**  **n (%)** | **Disagree**  **n (%)** | **OR (95%CI)** | **p-value** | **aOR (95%CI)** | **p-value** |
| **Total** | 45 (10.1) | 401 (89.9) |  |  |  |  |
| **Sex** |  |  |  |  |  |  |
| Female | 34 (10.1) | 303 (89.9) | 1 | (ref.) | 1 | (ref.) |
| Male | 11 (10.1) | 98 (89.9) | 1.00 (0.49-2.05) | 0.99 | 0.63 (0.28-1.45) | 0.28 |
| **Age** |  |  |  |  |  |  |
| ≤20 | 17 (16.3) | 87 (83.7) | 1 | (ref.) | 1 | (ref.) |
| 21-22 | 14 (8.8) | 146 (91.3) | 2.04 (0.96-4.34) | 0.07 | 2.14 (0.91-5.00) | 0.08 |
| ≥23 | 14 (7.7) | 168 (92.3) | **2.35 (1.10-4.98)** | **0.03** | **2.34 (1.03-5.34)** | **0.04** |
| **Discipline** |  |  |  |  |  |  |
| Medicine and Health | 14 (9.1) | 140 (90.9) | 1 | (ref.) | 1 | (ref.) |
| Arts and Social Science | 16 (15.7) | 86 (94.3) | 0.54 (0.5-1.16) | 0.11 | 0.86 (0.36-2.01) | 0.72 |
| Engineering & Food Science | 8 (6.5) | 116 (93.5) | 1.45 (0.59-3.58) | 0.42 | 1.71 (0.67-4.37) | 0.26 |
| Business and Commerce & Law | 7 (10.6) | 59 (89.4) | 0.84 (0.32-2.20) | 0.73 | 1.23 (0.43-3.49) | 0.70 |
| **Known someone** | 40 (9.9) | 365 (90.1) |  |  |  |  |
| Do not know anyone | 3 (5.4) | 53 (94.6) | 1 | (ref.) | 1 | (ref.) |
| Myself, partner, family or friends | 31 (10.6) | 262 (89.4) | 0.48 (0.14-1.62) | 0.24 | 0.47 (0.14-1.66) | 0.24 |
| Celebrities | 6 (10.7) | 50 (89.3) | 0.47 (0.11-1.99) | 0.31 | 0.49 (0.11-2.20) | 0.35 |
| **Rate of miscarriage** |  |  |  |  |  |  |
| Correct rate | 9 (9.8) | 83 (90.2) | 1 | (ref.) | 1 | (ref.) |
| Over-estimate fertility | 20 (12.0) | 146 (88.0) | 0.79 (0.35-1.82) | 0.58 | 0.81 (0.34-1.92) | 0.63 |
| Underestimate miscarriage | 16 (8.6) | 170 (89.9) | 1.15 (0.49-2.72) | 0.75 | 1.20 (0.48-3.03) | 0.69 |
